# Antibacterial photodynamic therapy: overview of a promising approach to fight antibiotic-resistant bacterial infections

**Published:** 2015-12-01

**Authors:** Yao Liu, Rong Qin, Sebastian A. J. Zaat, Eefjan Breukink, Michal Heger

**Affiliations:** 1 Department of Membrane Biochemistry and Biophysics, Utrecht University, the Netherlands; 2 Department of Medical Microbiology, Academic Medical Center, University of Amsterdam, the Netherlands; 3 Department of Experimental Surgery, Academic Medical Center, University of Amsterdam, the Netherlands

**Keywords:** Antibacterial photodynamic therapy, bacterial cell envelope, photosensitizer, non-selectivity, reactive oxygen species, singlet oxygen, multidrug resistance, light dose, illumination

## Abstract

Antibacterial photodynamic therapy (APDT) has drawn increasing attention from the scientific society for its potential to effectively kill multidrug-resistant pathogenic bacteria and for its low tendency to induce drug resistance that bacteria can rapidly develop against traditional antibiotic therapy. The review summarizes the mechanism of action of APDT, the photosensitizers, the barriers to PS localization, the targets, the in vitro-, in vivo-, and clinical evidence, the current developments in terms of treating Gram-positive and Gram-negative bacteria, the limitations, as well as future perspectives.

**Relevance for patients:** A structured overview of all important aspects of APDT is provided in the context of resistant bacterial species. The information presented is relevant and accessible for scientists as well as clinicians, whose joint effort is required to ensure that this technology benefits patients in the post-antibiotic era.

## Outline

1. Antibacterial photodynamic therapy in the ‘post-antibiotic’ era (141)
2. Photosensitizers for antibacterial photodynamic therapy (142)
  2.1. *Phenothiaziniums* (142)
  2.2. *Porphyrins* (146)
  2.3. *Phthalocyanines*(146)
  2.4. *Fullerenes* (147)
  2.5. *Naturally occurring photosensitizers* (147)
    2.5.1. *Hypericin* (147)
    2.5.2. *Curcumin* (147)
3. The barriers in antibacterial photodynamic therapy (147)
  3.1. *Lipopolysaccharides of Gram-negative bacteria* (147)
  3.2. *Outer membrane of Gram-negative bacteria* (150)
  3.3. *Teichoic acids of Gram-positive bacteria* (150)
  3.4. *Peptidoglycan* (151)
  3.5. *Cytoplasmic phospholipid bilayer membrane* (151)
4. The (putative) targets of antibacterial photodynamic therapy (151)
  4.1. *Lipids* (151)
  4.2. *Nucleic acids*(152)
  4.3. *Proteins* (152)
5. Targeting of pathogenic bacteria (152)
  5.1. *Pseudomonas aeruginosa (Gram-negative)* (152)
  5.2. *Staphylococcus aureus (Gram-positive)* (153)
  5.3. *Mycobacterium tuberculosis (Gram-negative)* (153)
  5.4. *Streptococcus mutans (Gram-positive)* (154)
  5.5. *Enterococcus faecalis (Gram-positive)* (154)
  5.6. *Helicobacter pylori (Gram-negative)* (155)
6. Optimization of photodynamic efficacy (155)
  6.1. *Liposomal photosensitizer delivery systems* (155)
  6.2. *Conjugation of cationic antimicrobial peptides to photosensitizers* (155)
  6.3. *Efflux pump inhibitors* (155)
  6.4. *Electroporation* (155)
  6.5. *Light source* (156)
7. In vivo and clinical status quo of antibacterial photodynamic therapy (156)
8. Adaptive mechanisms in bacteria and therapeutic recalcitrance (156)
9. Conclusions (157)
References(158)

## 1. Antibacterial photodynamic therapy in the ‘post-antibiotic’ era

The multidrug resistance of pathogenic bacteria has become a serious threat to public health. The World Health Organization released a report in April 2014 warning that the “post-antibiotic” era, “in which minor injuries and common infections can kill,” is approaching. Gram-positive and Gram-negative superbugs such as *Enterococcus faecium, Staphylococcus aureus, Klebsiella pneumoniae, Acinetobacter baumannii, Pseudomonas aeruginosa*, and *Enterobacter* species, the so-called “ESKAPE” pathogens, are capable of resisting almost all types/classes of antibiotics. Finding novel approaches to combat multidrug-resistant bacteria has therefore become increasingly important.

**Figure 1.**
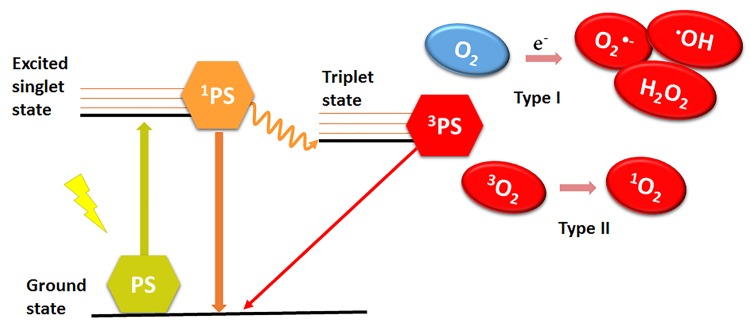
Type I and type II mechanism of ROS generation by photodynamic therapy. Abbreviations: PS, ground state photosensitizer; ^1^PS, photosensitizer in first excited state; ^3^PS, triplet state photosensitizer; e^‒^, electron; O2^•‒^, superoxide anion; •OH, hydroxyl radical; H_2_O_2_, hydrogen peroxide; ^3^O2, triplet state oxygen (molecular oxygen); ^1^O2, singlet oxygen.

Antibacterial photodynamic therapy (APDT) is a promising approach to treat bacterial infections that are recalcitrant to antibiotics. APDT is based on the photosensitization of bacteria with exogenous compounds referred to as photosensitizers (PSs). Cell death is subsequently triggered by lethal oxidative stress that is induced by irradiation of the infected area with light of a resonant wavelength, typically in the visible wavelength range (400-700 nm). The irradiated ground state PS, located in the bacteria or at the bacterial surface, absorbs the light and is excited to its singlet state (^1^PS). The excited state electrons undergo intersystem crossing to a lower-energy but longer-lived triplet state (^3^PS), from which reactive oxygen species (ROS) or reactive molecular transients are generated [1]. The photochemical reactions proceed via a type I or type II mechanism and require close proximity between the ^3^PS and substrate. Type I reactions generate radicals following triplet state electron transfer from the ^3^PS to a substrate. A common terminal substrate for type I reactions is molecular oxygen, leading to the production of superoxide anion (O_2_^•‒^). In a biological environment, O_2_^•‒^ is relatively innocuous but can give rise to more cytotoxic ROS such as hydroxyl radicals (•OH) and carbonate radical anions (CO_3_^•‒^) that oxidize biomolecules and cause cell damage and ultimately death [1-3]. In type II reactions, the excited PS reacts directly with molecular oxygen (O_2_) and forms the highly reactive singlet oxygen (^1^O_2_) via ^3^PS→O_2_ energy transfer. Type I and type II reactions (Figure 1) are believed to occur simultaneously during APDT and the ratio of the occurrence between the two is dependent on the type of PS that is administered and the microenvironment in which APDT is applied.

APDT has several advantages over antibiotics. The first important advantage is that APDT is considered triply site-specific due to (1) preponderant association/uptake of PSs by the target cells compared to non-target cells, (2) the pharmacodynamic inertia of non-irradiated PSs, as well as (3) the site-confined irradiation of the infected area. Consequently, (systemic) toxicity is largely absent outside the irradiated, PS-replete zone. Another important advantage of APDT over antibiotics is that no resistance is developed against the PSs. In that respect, repeated treatment with APDT did not lead to selection of resistant strains [4]. This is due to several reasons. First, the drug-light interval (the time between administration of the PS and PDT) is too short for bacteria to develop resistance. Second, PSs typically exhibit no dark toxicity, as a result of which bacteria do not have to engage adaptive survival mechanisms against the PSs. It is also difficult for bacteria to ‘sense’ that the oxidative stress emanates from the otherwise non-toxic PS, so any metabolic adaptations are directed elsewhere (e.g., antioxidant defense machinery). Third, the cells are too damaged after PDT, disabling them to confer cross-generation adaptivity. Lastly, APDT does not target a single site in bacteria, much different from conventional antibiotics. The ROS generated by APDT target various bacterial cell structures and different metabolic pathways [5]. These reasons underlie the potential utility of APDT in combatting resistant strains in a non-tominimally invasive and patient-friendly manner.

In light of the spread of resistant ESKAPE pathogens and the potential utility of APDT, this review summarizes the mechanism of action of APDT, the PSs used for APDT, PS pharmacokinetics and its cellular targets, the in vitro-, in vivo-, and clinical evidence for the utility of APDT, the status quo of APDT for both Gram-positive and Gram-negative bacteria, and potential strategies to optimize APDT. As the literature on APDT has expanded dramatically in recent years, the present review focuses mainly on the most recent information and novel developments. For more in-depth information on APDT, readers are referred to Hamblin et al. [6].

## 2. Photosensitizers for antibacterial photodynamic therapy

The efficacy of APDT relies chiefly on an optimal combination of PS and light. The ideal PS for APDT should exhibit high phototoxicity, low dark toxicity, high quantum yield of ^1^O_2_ or free radicals, preferential association with bacteria versus host mammalian cells, suitable pharmacokinetics, and accumulation in bacteria or binding to the bacterial cell envelope [7]. PS binding to the bacterial cell and uptake are dependent on the bacterial species. Due to the distinctive structure of the cell envelope, Gram-positive pathogens are much more susceptible to anionic and neutral PS because of the thick but porous peptidoglycan layer on the outer surface. Gram-negative bacteria are less prone to take up exogenous compounds due to the extra outer membrane and the permeability barrier imparted by lipopolysaccharides.

Preferably, APDT should be performed with cationic PSs in both Gram species. Cationic phenothiazinium-, phthalocyanine-, and porphyrin derivatives have been shown to significantly enhance phototoxicity in both Gram-positive and Gram-negative species [8-10]. It should be noted that, in some cases, negatively charged or neutral PSs at high concentration were more effective than cationic PSs [11,12]. Although not abundantly taken up by the bacteria, the PSs accumulate extracellularly in close proximity to the cell membrane and membrane constituents. The generation of reactive intermediates in close vicinity of cell structures either causes direct oxidation of these components or allows transmembrane diffusion of reactive intermediates and corollary oxidative damage to various intracellular targets [13]. In most instances, APDT predominantly proceeds via type II processes. However by comparing PSs that tend to undergo either type I or type II mechanism, Huang et al. reported that Gram-negative species are more susceptible to •OH than ^1^O_2_ [1]. A type I reaction is therefore favored when targeting Gram-negative species.

**Figure 2.**
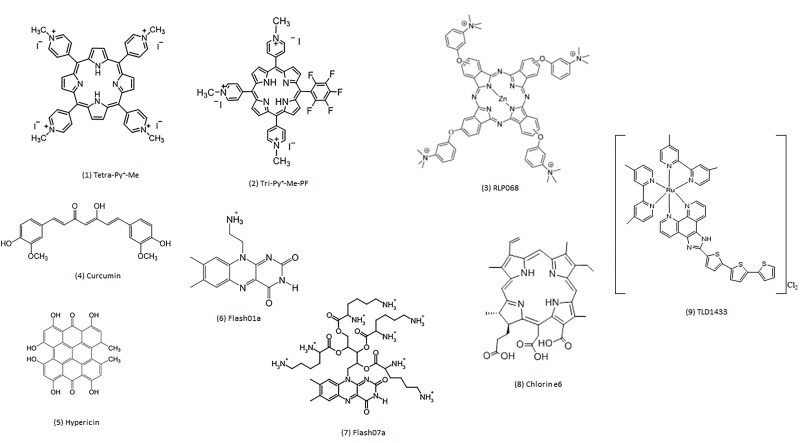
The structure of some photosensitizers covered in this review. Phenothiazinium photosensitizers are listed separately in Table 2.

The most extensively studied classes of PSs and their physical and chemical properties are presented in Table 1. The generic structure of each PS class is shown in Figure 2. Many PSs initially exhibited high inactivation efficacy against Gram-positive bacteria in their native form. However, these PSs were later modified structurally (e.g., addition of cationic functional groups) to improve therapeutic efficacy in Gram-negative species.

### 2.1. Phenothiaziniums

Phenothiazinium and its derivatives (Table 1), which include methylene blue (reviewed in [14]), Rose Bengal, and toluidine blue O, are a class of first-generation PSs that were initially investigated for PDT of solid cancers. These PSs are commonly employed in APDT because of their high binding affinity for both Gram-positive and Gram-negative bacteria such as methicillin-susceptible and methicillin-resistant *S. aureus* (MSSA and MRSA) and *E. coli* [15,16]. Phenothiazinium PSs are pharmacodynamically interesting because this class of PSs exhibits inherent toxicity to *E. coli* cells, also under dark conditions [17]. With respect to PDT, it has been shown that phenothiazinium targets cytoplasmic DNA [18].

**Table 1.**
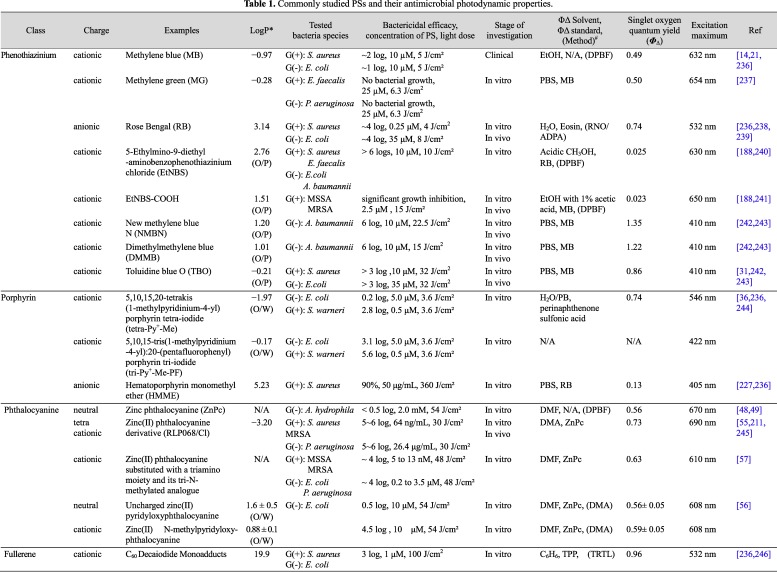

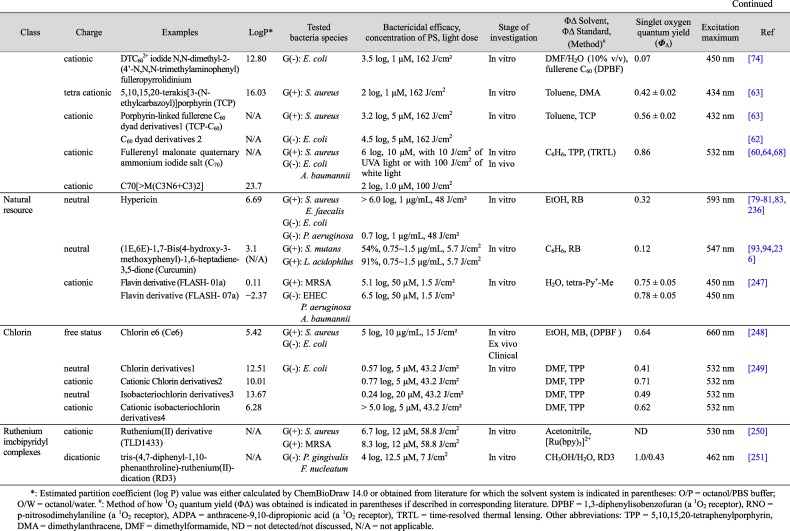
Commonly studied PSs and their antimicrobial photodynamic properties.

Methylene blue derivatives (structures are summarized in Table 2) such as new methylene blue, dimethyl methylene blue, and methylene green were developed with different functional substituents and shown to have improved effectiveness of killing bacteria. As indicated previously, increasing the positive charge on methylene blue correlated positively with increased APDT efficacy than native methylene blue [19,20], mainly because substitution with cationic functional groups improved the binding and uptake of the PS by bacteria relative to the parent structure [19-21]. For example, Felgentrager et al. revealed that derivatization of methylene blue with tertiary ammonium substituents increased the uptake by microbial cells due to the fact that these substituents imposed a greater cationic charge in a pH-equilibrated aqueous solution, and therefore more avid bacterial cell binding, compared to primary or secondary substituents [19]. Furthermore, the tertiary ammonium-substituted methylene blue derivative possesses lower pK_a_ values than the lower-order substituents, which results in faster deprotonation when bound to the negatively charged bacterial cell envelope structure and better uptake. It was further shown that, by employing relatively short drug-light intervals, APDT of the infected area killed the photosensitized bacteria without damaging the surrounding healthy cells/tissues [22-24].

**Table 2.**
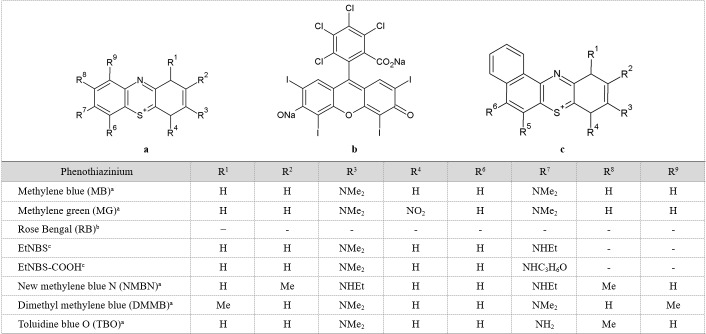
Structures of common phenothiazinium photosensitizers

Phenothiazinium is also a substrate for multidrug resistance pumps [25,26], a family of transmembrane proteins that mediate the efflux of amphipathic cations, amongst others. Multi-drug resistance pump inhibitors may therefore be employed when phenothiazinium-APDT yields suboptimal results. For example, Tegos et al. have shown that co-incubation of toluidine blue O with different efflux pump inhibitors (EPIs) such as NorA and Mex-AB increased the bactericidal effect of toluidine blue O by at least 2 logs in both *S. aureus* and *P. aeruginosa* [27]. It is noteworthy that this effect was more prominent when the EPI was administered before adding toluidine blue O than after, indicating that toluidine blue O competitively binds the pump binding site of EPI and therefore blocks its access when the EPI inhibitor is administered after incubation with toluidine blue O. Nevertheless this approach must be further investigated given that EPIs have not yet reached clinical development due to their high toxicity [26,28,29].

Another class of compounds that potentiates APDT with phenothiazinium or its derivatives is electron acceptors. A good example is sodium azide. Although sodium azide is a ^1^O_2_ quencher, the addition of sodium azide (10 mM or 0.1 mM) increased the bactericidal effect of MB-APDT (25 μM) in *E. coli* and *S. aureus* by > 1 log when illuminated with red light (620-750 nm), even in the absence of oxygen [30]. The adjuvant effect of sodium azide was attributed to the production of azidyl radicals. Inasmuch as such an effect was absent in chlorin(e6)-APDT [30], which produces mainly ^1^O_2_ through type II reactions [1], this modality requires the use of type I PSs to enable a one-electron oxidation of the azide anion by photo-activated PS. However, the exact mechanism of how the excited PS removes the electron from the azide anion requires further investigation. Similar results were obtained with other phenothiazinium-based PSs, including methylene blue, dimethyl methylene blue, new methylene blue, toluidine blue O, azure A, and azure B [31].

### 2.2. Porphyrins

Porphyrins (Table 1) are commonly employed for (A)PDT because of their high molar absorptivity, relatively high triplet state quantum yield [32], easy synthesis [33,34], and chemical versatility (i.e., easily modifiable). The photodynamic action of porphyrins stems from mainly a type II mechanism [35] following excitation with light of typically 405-550 nm.

The number of charges carried by a porphyrin derivative is positively correlated with its bactericidal efficacy, whereby charge distribution also plays a decisive role [36,37]. A cationic charge is a critical factor in APDT of Gram-positive species. Porphyrins with a net cationic charge, such as meso-substituted cationic porphyrins, effectively kill Gram-negative bacteria because of increased binding/uptake efficacy [38]. Corroboratively, two novel cationic porphyrins carrying pyridinium (PyP, Table 1) and imidazolium (ImP) substituents were shown to bind different loci at the bacterial outer membrane via ionic interactions, penetrate the cell wall, and enter the cell [39]. The PSs were able to reduce the viability of both Gram-positive and Gram-negative bacteria by 6 log with a PS concentration of 2 μM (ImP) or 6 μM (PyP) and a light dose of 60 J/cm^2^ [39].

In case of Gram-negative species, a high number of positive charges asymmetrically distributed across the structure as well as a hydrophobic *meso*-substituent group (e.g., tri-Py^+^-Me-PF, Table 1) are important determinants for PS-bacterial cell association [8,36,40]. Accordingly, the amphiphilicity of porphyrins dictates PS-cell interactions with respect to Gram-negative bacteria and hence the APDT outcome. The hydrophobic domain may undergo lipophilic interactions with the bacterial cell wall, whereas adjacent charges on the porphyrin promote electrostatic interaction with the negatively charged cell envelope and improve uptake [35,41]. To identify the PDT mechanism that underlies these amphiphilic PSs, Tavares et al. [35] compared the Gram-negative pathogen killing effects of tetra-Py^+^-Me, tri-Py^+^-Me-PF, and tri-SPy^+^-Me-PF in the presence of different ROS scavengers that tend to neutralize either the free radicals or the ^1^O_2_. The authors found that these porphyrin derivatives are likely to undergo a type II mechanism and produce ^1^O_2_.

Anionic and neutral porphyrins are generally not recommended for targeting Gram-negative bacteria due to their poor binding efficacy. However, the association of anionic and neutral porphyrins with Gram-negative bacteria can be considerably improved by the conjugation of cationic antimicrobial peptides (CAMPs) or cell penetrating peptides (CPPs) such as apidaecin [42,43], Tat [44], buforin, or magainin [45] to the PS. These PS-peptide conjugates effectively photosensitized *E. coli* and induced considerable cell death following APDT compared to unconjugated PS. Other examples of eligible antimicrobial peptides are described in [46].

It should be noted that porphyrins are also taken up by mammalian cells [47]. Unspecific targeting can therefore not be ruled out and may impair the selectivity of APDT.

### 2.3. Phthalocyanines

As porphyrins, phthalocyanines (Table 1) are heterocyclic macrocycle aromatic compounds (Table 1). Phthalocyanines differ from porphyrins by the isoindole subunits that are interconnected by a secondary amine bridge (versus methene bridge-interconnected pyrroles) [7], shifting the excitation maximum to longer wavelengths (typically > 660 nm). The mostly studied phthalocyanines for APDT are based on zinc (II) phthalocyanine (ZnPc) [9,48-54]. ZnPc predominantly generates ^1^O_2_ upon excitation [54].

Native ZnPc was found to be ineffective against Gram-negative bacteria such as *E.coli* [48] and had to be applied in combination with membrane perturbing agents such as ethylenediaminetetraacetic acid (EDTA) or calcium chloride to ensure (intra)cellular delivery [23,55,56]. In contrast, ZnPc exhibited affinity for the Gram-positive *Streptococcus mitis*, which involved the association with membrane proteins residing in the cytoplasmic membrane [49,54]. Later studies revealed that the introduction of a positive charge considerably improved the binding affinity and inactivation efficacy in Gram-positive bacteria, but also Gram-negative bacteria such as *E. coli* and *Pseudomonas aeruginosa* [9,57]. Illustratively, Spesia et al. [53] discovered that cationic ZnPc was able to eliminate *E.coli* in the presence of human blood derivatives. This finding spawned the possibility that APDT may be used for blood product sterilization as a result of selective bacterial membrane targeting, while preventing hemolysis stemming from erythrocyte membrane oxidation and rupture.

Subsequent efforts were directed at optimizing this class of PSs. While studying 14 anionic, cationic, or neutral zinc and aluminum-containing phthalocyanine derivatives, Mikula et al. [58] found that, of all cationic phthalocyanines that exhibited particularly high antibacterial activity and low dark toxicity, those with at least one amino group in the substituent bound with greatest affinity to the negatively charged surface of the *E. coli* outer membrane. The authors attributed the bonding behavior of the amino group to the high positive charge density.

To date, investigations on the use of phthalocyanines for APDT comprise in vitro studies in buffer or solution. The in vivo use and clinical relevance have not yet been addressed experimentally.

### 2.4. Fullerenes

Spheroidal fullerenes such as C60 [59] and C70 [60] (Table 1) are football-like structures composed of pentagonal and hexagonal rings that can absorb visible light [61] and mediate photochemical reactions from the excited state [60,62-64]. The photo-excited fullerenes can react with electron donors to generate fullerene radical anions (C60^•−^, C70^•−^) [61,65-68], which in turn can mediate the production of •OH in the presence of O_2_. These properties make fullerenes suitable candidates for APDT because bacteria can simultaneously provide a number of electron donors from organic sources (NADH and succinate [69]) and inorganic sources (hydrogen [70], ammonia [71], sulfur [72], and ferrous iron [73]).

Due to the highly lipophilic and non-charged structure, native fullerenes exhibit poor PS-bacterial cell association and are therefore relatively inactive as PSs against bacteria [64,74]. Fullerenes must be chemically modified with amphiphilic molecules [75] to enable bacterial photosensitization. It has been demonstrated that the derivatization translates to high selectivity towards bacterial cells [59]. Accordingly, a cationic fullerene derivative N,N-dimethyl-2-(40-N,N,N-trimethylaminophenyl)fulleropyrrolidinium iodide (DTC60^2+^) [74] considerably enhanced APDT efficacy of *E. coli* and *Pseudomonas aeruginosa* (> 3.5 log) compared to the negligible killing effect with non-charged N-methyl-2-(40-acetamidophenyl) fulleropyrrolidine (MAC_60_). Moreover, Hamblin et al. [76] showed that, when iodide was added as an electron donor, cationic C60-fullerene (LC16) produced •OH through a type I mechanism and effectively killed *Acinetobacter baumannii* and MRSA. Another study published by the same group [77] showed that azide can also act as an electron donor in a similar fashion. Finally, fullerene derivatives are more stable than tetrapyrrole-based PSs such as porphyrins and chlorins [74], which may render these PSs more suitable for pharmacological formulations and clinical use. A dedicated review on fullerenes as potent PSs in PDT and APDT is available elsewhere [64].

### 2.5. Naturally occurring photosensitizers

#### 2.5.1. Hypericin

The most commonly used naturally occurring PS is hypericin (Table 1), a red-colored anthraquinone derivative that comprises one of the main bioactive constituents of Saint John’s wort (*Hypericum*). The PS, with an excitation maximum at 593 nm, exhibits anti-cancer, anti-viral, and anti-bacterial properties that, in case of viruses and bacteria, have been ascribed to its photosensitization effects [78-81]. Hypericin confers phototoxicity through a type II mechanism in MSSA and MRSA as well as pathogenic *E. coli* [80,82,83]. Maximum ROS generation by hypericin and its derivatives is achieved in a lipophilic milieu [84].

Hypericin-mediated phototoxicity was found to be more profound in planktonic *S. aureus* than in its biofilm-forming counterpart [82]. This is possibly due to the presence of polysaccharide intercellular adhesin (PIA) in the biofilm that blocks the uptake of hydrophobic PSs such as hypericin [85]. Photokilling of bacteria correlates with the time of pre-incubation with hypericin; longer pre-incubation (24 hours) led to more *S. aureus* cell death than shorter pre-incubation periods [80].

A good method to improve APDT of biofilm-producing bacteria is chemical perturbation of the biofilm. N-acetylcysteine is a highly suitable adjuvant, as it breaks down the bacterial biofilms of clinically relevant pathogens, including *S. aureus*, *Pseudomonas aeruginosa*, *Enterococcus faecalis*, and *Staphylococcus epidermidis* [86,87]. Correspondingly, Kashef et al. [82] demonstrated that the combined use of hypericin and N-acetylcysteine increased intracellular delivery of hypericin in *S. aureus*. The joint application of hypericin with N-acetylcysteine therefore warrants further investigation, although it should be kept in mind that N-acetylcysteine is a glutathione precursor [88,89] and may therefore strengthen the bacterial antioxidant machinery in the hydrophilic compartment, and thereby reduce APDT efficacy. This holds particularly for Gram-negative species since glutathione synthesis is not found in most Gram-positive species [90].

#### 2.5.2. Curcumin

Another naturally occurring PS is curcumin (Table 1) [91], a yellow pigment derived from the root of the *Curcuma longa* plant [92,93]. Curcumin is a polyphenolic compound that absorbs visible light in the 405-435-nm range, depending on the chemical environment [94]. Due to its unique chemical properties, curcumin exhibits pleiotropic binding towards many types of biomolecules, including (phospho)lipids, proteins, and nucleic acids [94].

The photokilling propensity of curcumin was considerably more profound in the Gram-positive *S. aureus* than the Gram-negative *E. coli* and *Salmonella typhimurium* (~300 times more effective when corrected for light dose and concentration) [95], indicating an associative predilection for Gram-positive species. The bactericidal effect of light-irradiated curcumin occurs at relatively short drug-light intervals (0-60 min), whereby longer drug-light intervals (90 min) did not yield an additional cytotoxic effect [95]. These data suggest that curcumin either distributes rapidly throughout the cell or confers its phototoxic effect at superficial loci, from which the generated radical intermediates may induce local damage or diffuse to intracellular targets. At this point, however, the cytotoxic mechanisms in curcumin-APDT-subjected bacteria are elusive. The production of ROS by light-irradiated curcumin has been exclusively studied in the context of mammalian cells [96] but not bacterial cells. In rat basophilic leukemia cells it was shown that carbon-centered radicals with a long lifetime (up to 27 seconds) were produced that may have been responsible for the phototoxicity of curcumin. These findings may explain why surface binding of curcumin could suffice to kill bacteria, given that these intermediates are more likely to transgress the cell wall than the short-lived ^1^O_2_ or •OH [13].

A contributory mechanism to cytotoxicity is that curcumin generates radicals or become a radical itself in a physiological environment in the absence of light, especially at pH > 6.5 [94]. On the other hand, curcumin is also very photo-labile [94], as a result of which its photodynamic action may be rather short-lived due to rapid photodegradation. In any case, the use of curcumin as a PS deserves further investigation, particularly since the molecule can be modified chemically at the flanking or central functional groups [94] without perturbing the conjugated system required for triplet state generation. The conjugation of cationic moieties may further enhance curcumin’s APDT efficacy.

## 3. The barriers in antibacterial photodynamic therapy

Traditional antibiotics often utilize a key-hole mechanism, where the compounds target one specific membrane- or (intra)cellular component in bacteria, be it proteins, lipids, or DNA, to either stop growth or kill the organism. For example, penicillin binds to the penicillin binding proteins and inhibits the crosslinking of the peptidoglycan multi-layer [97]. Vancomycin binds to the D-Ala-D-Ala residues of the peptide side chain of the peptidoglycan precursor lipid II and deters downstream peptidoglycan synthesis steps [98]. Daptomycin is believed to insert into the membrane of Gram-positive bacteria, where it forms aggregates that modify the curvature of the membrane and cause cavitation, ion leakage, and ultimately cell death [99].

**Figure 3.**
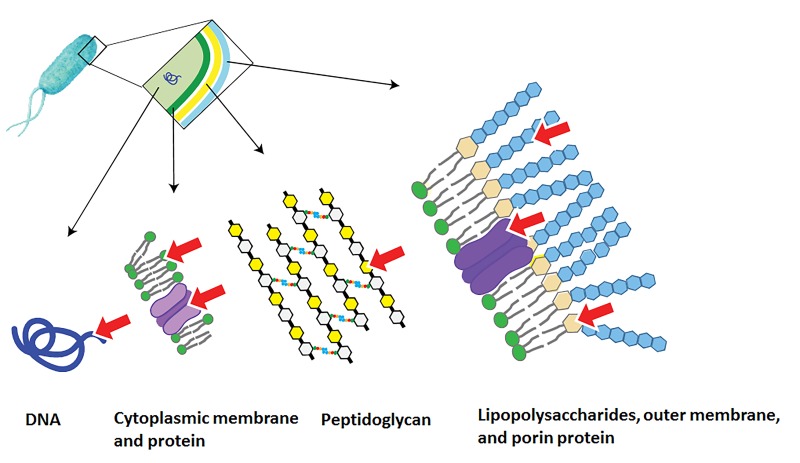
Overview of APDT targets in bacterial cells, indicated by a red arrow.

In contrast, the PSs used for APDT typically distribute to multiple extracellular or intracellular compartments and/or produce radical intermediates that can migrate away from the formation site. As a result, various components of cell metabolism are disrupted, culminating in cell demise when sufficiently afflicted. The extracellular and intracellular targets of the commonly used PSs are summarized in Table 3 and a generic overview of possible target loci is provided in Figure 3. For a more detailed overview of the targets, readers are referred to a recent review [100]. Since APDT efficacy largely depends on the localization of the PS, the following subsections focus of the physical and biochemical hurdles that impair the PSs in reaching the target sites.

The inactivation of bacteria is dependent on the association of the PS with the pathogen. The first step is the binding or interaction of the PS to the bacterial cell surface. Both Gram-positive and Gram-negative bacteria have an overall negatively charged cell surface comprised of different surface structures [101]. The anionic surface therefore acts as an electroattractive scaffold for cationic PSs, which are more efficiently bound to and taken up by bacteria [102]. Due to the short lifetimes of most radical intermediates, and particularly of type II-generated ^1^O_2_ and type I-produced •OH, it is preferable that the PS is taken up for maximum oxidative damage following APDT. Owing to the complexity of the bacterial cell envelope, the uptake of PSs is, however, hampered by several factors addressed next. Readers should note that in practice, the exclusion of exogenous compounds from the intracellular space is not attributable to a single barrier acting on its own, but rather in concert with other barriers. Potential means to overcome these barriers are also discussed where applicable.

### 3.1. Lipopolysaccharides of Gram-negative bacteria

**Figure 4.**
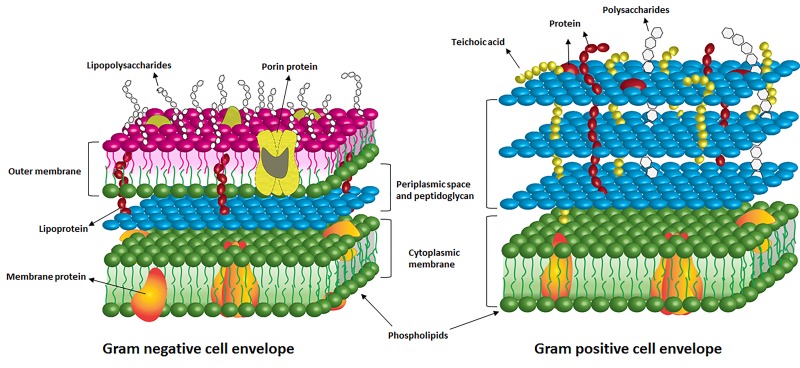
Illustration of the cell envelope of Gram-negative and Gram-positive bacteria.

Lipopolysaccharides (LPS) are the major component of the outer membrane of Gram-negative bacteria (Figure 4) that impart structural integrity and protect the membrane from attacks by chemicals. LPS forms the outermost physical and electrostatic barrier that exogenous compounds must transgress to reach the lipid bilayer of the outer membrane. Its presence is therefore a hurdle for intracellular PS targeting. Although the LPS layer obstructs easy entry of PSs into Gram-negative bacteria, the layer may also serve as a target for APDT [18,103]. The surface structures are vitally important in bacterial cell physiology. To underscore the importance of LPS: when LPS is structurally modified or removed, the bacteria die.

Because of its highly anionic nature, LPS is considered a primary target of cationic PSs. A cationic charge is not the only determinant governing PS association with LPS, as toluidine blue O (logP = –0.21) exhibited higher affinity towards LPS isolated from different bacterial strains than MB (logP = –0.97) [18], while both PSs have a formal charge of 1. LPS as a target also serves another clinical purpose, namely to activate the innate and adaptive immune system of the host organism [104], which may aid to an extent in post-APDT removal of residual pathogens [105,106].

**Table 3.** The extra- and intracellular targets of some common photosensitizers

Class	Name	Extracellular target	Intracellular target	Bacteria	Ref.
Phenothiazinium	Methylene blue (MB)	Cell wall surface and membrane protein	Chromosomal DNA	*E. faecalis*	[252]
	Rose Bengal (RB)	Cytoplasmic membrane	DNA*	*E. coli*	[253]
	Toluidine blue O (TBO)	Lipopolysaccharides and outer membrane	ND	*P. aeruginosa*	[254]
Porphyrin	5,10,15,20-tetrakis(1-methylpyridinium-4-yl)porphyrin tetra-iodide (Tetra-Py^+^-Me)	Lipopolysaccharides and outer membrane lipids	DNA*	*E. coli, Aeromonas salmonicida, Aeromonas hydrophila, Rhodopirellula sp,*, *S. aureus, Truepera radiovictrix, Deinococcus geothermalis, Deinococcus* *radiodurans*	[114,127]
	5,10,15,20-tetra(4-N,N,N-trimethylammo-niumphenyl) porphyrin	Cell wall and cytoplasmic membrane	Plasmid DNA	*E. coli*	[41]
	5,10,15,20-tetrakis(N-methyl-4-pyridyl): 21H,23H-porphine (Tetra-Py^+^-Me)	Outer membrane	ND	*E. coli*	[124]
	Hematoporphyrin monomethyl ether (HMME)	Cytoplasmic membrane	ND	*S. aureus*	[255]
Phthalocyanine	Zinc(II) phthalocyanine (ZnPc)	Outer membrane and cytoplasmic membrane	ND	*E. coli*	[48]
Fullerene	N-methylpyrrolidinium C_60_ fullerene iodide salt	Cytoplasmic membrane	ND	*S. aureus*	[256]

* DNA as target of APDT still requires further investigation. In most studies, it is not distinguished whether the DNA damage comprises chromosomal DNA or plasmid DNA; ND = not detected/not discussed.

In regard to LPS redox modification by APDT, a recent study [107] reported that chitosan-conjugated Rose Bengal nanoparticles and methylene blue effectively neutralized LPS isolated from *P. aeruginosa* and reduced the inflammatory potency of LPS. Given that selective inactivation of LPS is not associated with detrimental side effects in mammalian host cells, such PSs should be further explored for their utility in a clinical setting, where the infected tissue is in close proximity to healthy tissue. Moreover, the binding of the PS to LPS competes with cations such as Ca^2+^ and Mg^2+^ that stabilize the bacterial membrane structure and integrity. Displacement of structurally important cations weakens the outer membrane and leads to a ‘self-promoted’ pathway of PS uptake, as has been documented in [103]. Although a cationic PS (poly-L-lysine chlorin(e6) conjugate, pL-e6) binds 15-100 times more efficiently to *S. aureus* than to *E. coli*, the killing effect was much stronger in the latter. This is mainly ascribed to the fact that divalent cation replacement in *E. coli* LPS by the cationic PS led to membrane distortion and pore formation to promote uptake. In contrast, the thick peptidoglycan of *S. aureus* blocked such access, although the PS bound extensively to the negatively charged cell surface [108].

### 3.2. Outer membrane of Gram-negative bacteria

The outer membrane is comprised of a phospholipid bilayer (Figure 3) whose barrier function is typical of all biomembranes. Biomembranes have hydrophilic surfaces and lipophilic cores, whereby the hydration state of the membrane (and thus penetrability of water-soluble molecules) decreases towards the midplane of the bilayer [109]. This chemical composition of the bilayer makes the outer membrane as well as the cytoplasmic membrane a very effective barrier against a vast number of molecules [109]. Typically, very lipophilic molecules (high logP, e.g., phthalocyanines, section 2.3) get trapped in the bilayer, whereas very hydrophilic molecules (low logP) cannot pass through the bilayer core due to chemical incompatibility. Because the tightly packed component phospholipids do not leave much room for interposition, large molecules (e.g., fullerenes, section 2.4) are also excluded. Only amphipathic molecules, i.e., molecules with lipophilic backbones and polar/charged flanks (medium logP, e.g., curcumin and hypericin, section 2.5) are quite capable of transgressing membranes [94].

Despite the evolutionary perfected barrier function of biomembranes, the structures are not impervious. Disruption of LPS upon displacement of divalent ions, as described in the

previous section, results in weakening of the outer membrane as a result of which the outer membrane becomes accessible to PSs [103]. The uptake of cationic PSs across the outer membrane subsequently proceeds via a so-called self-promoted pathway [103]. This pathway entails the replacement of important LPS-binding divalent ions (Ca^2+^ and Mg^2+^) by cationic PS, thereby disrupting outer membrane stability, leading to the formation of channels in the outer membrane that can facilitate PS uptake.

The self-promoted uptake is not as straightforward for anionic PSs. Anionic PSs may require an active transport machinery [108]. Porins, which are transmembrane proteins situated in the outer membrane of Gram-negative bacteria [110,111], are possible candidates insofar as these proteins are responsible for the transport of substances such as sugars, small peptides, and drugs [111]. It has been shown that the degree of uptake of some anionic PSs (Rose Bengal and indocyanine green) decreased following trypsin treatment, suggesting the involvement of protein transporters in shuttling PSs across the outer membrane [108]. In contrast, the uptake of MB (cationic) was not affected by trypsin [48]. Taken together, the outer membrane is a second barrier to cellular entry of exogenous compounds, albeit one that can be circumvented in case of both anionic and cationic PSs. In case of neutral PSs, conjugation of the PS to a cationic molecule could sufficiently facilitate outer membrane penetration by the PS [57].

### 3.3. Teichoic acids of Gram-positive bacteria

Comparable to Gram-negative LPS, teichoic acids in Gram-positive species (Figure 3) contribute to the net negative charge on the bacterial cell surface. The carboxylate and phosphate groups of teichoic acids (comprising the phosphodiester bonds between teichoic acid monomers) are responsible for the negative charge of these acids [112].

Teichoic acids including lipoteichoic acids that are attached to the cytoplasmic membrane and wall teichoic acids that are bound to the peptidoglycan impart essentially all of the negativity of the peptidoglycan/periplasmic space of Gram-positive bacteria [112]. The anionic residues are also a major binding site of divalent ions such as Mg^2+^ and Ca^2+^ [101], altogether accounting for an electrostatic barrier for mainly anionic molecules. Cationic PSs can get passed these residues through electrostatic interactions, while anionic PSs are repulsed by the negative charges [108]. However, as for LPS, the electrostatic interaction between structural cations and cationic PSs is competitive [103], rendering the membrane surface biochemically ideal for cationic PSs. Also, deionization of the outer membrane surface (Gram-negative bacteria) or the peptidoglycan (Gram-positive bacteria) will result in destabilization of the barrier function and facilitate PS passage [108].

### 3.4. Peptidoglycan

As shown in Figure 4, the peptidoglycan is situated on the outer surface of the cytoplasmic membrane in Gram-positive bacteria and between the outer membrane and cytoplasmic membrane in Gram-negative bacteria. Being the outer most layer in Gram-positive bacteria, the peptidoglycan layer is 2-10 times thicker (20-80 nm) in these cells than in Gram-negative bacteria to enable optimal barrier function. The average hole size of peptidoglycan was found to be 2.06 nm for the Gram-negative *E. coli* and 2.12 nm for Gram-positive *Bacillus subtilis* [113]. Due to the structural and anatomical differences, the degree of physical and chemical fortification imposed by the peptidoglycan in Gram-negative bacteria may be less significant than in Gram-positive bacteria.

In both Gram species, however, the peptidoglycan is a relatively porous layer and rather inadequate in terms of a permeability barrier for most PSs [13]. Even neutral and anionic PSs, which should be repulsed by the negatively charged peptidoglycan, are under certain circumstances able to diffuse through this multi-layered scaffold [108]. Pereira et al. [114] reported that the uptake and APDT efficacy of cationic porphyrins are closely related to the chemical composition of the external structures of different bacteria. Gram-positive bacteria, even with a more complex and multilayered envelope (*T. radiovictrix, D. geothermalis*, and *D. radiodurans* ) showed higher susceptibility towards porphyrin derivatives (Tetra-Py-Me^+^). These findings notwithstanding, the permeability of the peptidoglycan make Gram-positive bacteria more suitable targets for APDT compared to Gram-negative bacteria.

### 3.5. Cytoplasmic phospholipid bilayer membrane

The barrier function of biomembranes was explained in section 3.2 and also applies to the cytoplasmic membrane. However, the differential composition of the cytoplasmic membrane between Gram-positive and Gram-negative bacteria does dictate the degree to which this membrane exerts a barrier function. The zwitterionic phospholipid phosphatidylethanolamine constitutes the most abundant phospholipid in bacteria, but is more replete in Gram-negative strains than in Gram-positive bacteria [115]. All bacteria, regardless of the gramspecies, have at least 15% anionic lipids comprising either phosphatidylglycerol, cardiolipin, or both [116,117]. The cytoplasmic membrane of some Gram-positive bacteria has low phosphatidylenthanolamine content and is, by ratio, therefore enriched with more anionic phospholipids [115]. This, along with other factors addressed in the previous subsections, explains why the uptake of many cationic PSs is more prolific in Gram-positive species than their Gram-negative counterparts. Consequently, APDT is more effective in Gram-positive bacteria. This is of particular interest when considering that the drug-resistant pathogenic Gram-positive strains such as MRSA and vancomycin-resistant enterococci species, among others, are a real threat to public health [118].

## 4. The (putative) targets of antibacterial photodynamic therapy

### 4.1. Lipids

Owing to their lipophilicity, most PSs (Table 1) localize to cellular and subcellular membranes following uptake [13]. It therefore follows that lipids are an important target for APDT [114] and that the phototoxicity induced by APDT emanates from oxidative modification of component lipids and membrane proteins (Figure 4) that, just as LPS, are vital to cell physiology and metabolism. The ROS and other radical intermediates mainly target the double bonds in the phospholipid acyl chains, i.e., unsaturated fatty acids [119]. Accordingly, the bacterial strains in which the outer membrane leaflet is composed of saturated fatty acids or a relatively small fraction of unsaturated fatty acids are less susceptible to APDT [120]. Another study [121] reported that the phospholipids in the inner leaflet of the outer membrane and the cytoplasmic membrane of *E. coli* are a target of redox modification induced by a porphyrin derivative (tri-Py^+^-Me-PF). The extensive formation of lipid hydroperoxides and a decrease in the amount of unsaturated fatty acyl chains were observed in lipid extract as a result of APDT.

Oxidative modification of membrane (phospho)lipids alters membrane fluidity and organization as well as membrane protein function [119] that, when extensive enough, culminate in cell death [115,122]. Outer membrane targeting for APDT should therefore be sufficient to induce massive cytotoxicity and circumvent the hurdles associated with intracellular delivery of the PSs (section 3). Some proof-of-concept has already been provided. Johnson et al. [123] studied PS localization and APDT efficacy using eosin-(KLAKLAK)_2_ as PS-peptide conjugate prior to APDT of *E. coli* and *S. aureus*. Their results revealed that the PS-peptide complex localized to the cell surface. The (KLAKLAK)_2_ initially acted as a targeting ligand and, after eosin-induced lipid oxidation had occurred following APDT, as a membrane perturbing agent. The cumulative effect, which was facilitated by redox modification of lipids, was bacterial cell death in the absence of intracellular PS accumulation.

Some porphyrin derivatives have also been reported to exhibit membrane accumulation and induce site-confined lipid peroxidation. Two studies [114,124] have demonstrated that short incubation (1-3 hours) of Gram-negative bacteria with tetra-Py^+^-Me (Table 1) led to PS accumulation in the outer membrane without intracellular accumulation. Longer incubation times result in redistribution of tetra-Py^+^-Me into the cell. In *E. coli*, Ragas et al. [125] showed that tetra-Py^+^-Me localized to the outer surface as well as intracellularly following 20 hours of incubation. Accordingly, the production of ^1^O_2_ occurred at multiple locations.

### 4.2. Nucleic acids

Whether nucleic acids are a primary target of APDT is presently elusive and marked by controversial data. Several studies have suggested the binding of PSs to nucleic acids [125,126], which implies that nucleic acids are oxidatively modified by APDT and induce genetic catastrophe after treatment. Support for this hypothesis was provided by an APDT study with the hydrophilic cationic porphyrins tetra-Py^+^-Me and tri-Py^+^-Me-PF (Table 1), which demonstrated that APDT-induced nucleic acid damage occurred in the order of 23S rRNA > 16S rRNA > DNA in *E. coli* and *S. warneri* [127]. In contrast, Samon-Divon et al. [128] reported that DNA damage occurs first following APDT with tetra-Py^+^-Me, at least in *E. coli*. Extensive production of ^1^O_2_ and the cell permeation properties of these PSs were cited to be responsible for the nucleic acid damage that occurred inside the bacterial cells [35].

Nevertheless, whether DNA or RNA is the primary target of APDT remains an open debate [127,129,130]. For example, a proteomics study aimed at identifying the molecular targets of APDT of MRSA using tetra-Py^+^-Me [131] concluded that nucleic acids are not likely a primary target because DNA and RNA damage can be repaired by various repair pathways. It was reported that RecA expression in *S. aureus*, which triggers the SOS signal for DNA repair, is upregulated during APDT. This mechanism does not seem to be ubiquitous, however, as similar results were not found in *P. aeruginosa* [132]. So although nucleic acids may be oxidized as a result of APDT, the redox modifications can be reverted and the cells may not undergo cell death as a direct result of nucleic acid damage. Moreover, DNA damage was only found when a remarkable amount of bacterial cells had been photo-inactivated, pleading against this mechanism as a primary cause of cell death [100]. More focused studies are needed to elucidate the role of nucleic acid oxidation in the context of cytotoxicity.

### 4.3. Proteins

Proteins are present in all compartments of the bacterial cell, from the surface (porin proteins, membrane proteins, lipoproteins) to the cytosol (soluble proteins) [133], and are important for various biological activities that occur in bacterial cells. Membrane proteins are likely the preferred targets for the PSs due to their shared lipophilicity.

Proteins are unequivocally damaged by APDT, although bacterial proteins are less sensitive to oxidative modification compared to mammalian proteins [114]. However, whether this damage translates to bacterial cell death is currently unclear. A protein-electrophoresis study performed in APDT-subjected *E. coli* and *S. warneri* using tetra-Py^+^-Me and tri-Py^+^-Me-PF showed that substantial protein degradation and a consequent decrease in cell viability occurred in both strains quite quickly after irradiation (5-60 min) [134]. Tri-Py^+^-Me-PF-APDT was more cytotoxic in both strains due to its higher ^1^O_2_ production. The authors suggested that the asymmetric structure and charges of tri-Py^+^-Me-PF as well as the lipophilic pentafluorophenyl group increased the binding affinity to bacterial cells, which has been echoed by other studies [35-37].

The rapid degradation of proteins corroborates redox modification following APDT, but does not provide information on whether this process accounts for or contributes to cell death. In fact, bacterial cells possess several coping mechanisms for proteotoxic stress [135], and the same argument may apply for proteins as for nucleic acids (i.e., initial oxidative damage, but no biological consequences due to repair). The proteomics study discussed in section 4.2 revealed an altered expression of proteins involved in carbohydrate uptake, cell division, and response to oxidative stress [131], whereby the latter response seems to be ubiquitous across multiple species, including human cancer cells [136]. Consequently, the cytotoxicity of APDT-induced proteotoxic stress requires closer investigation, also in light of the coping mechanisms and possible interventions to block the related survival pathways.

## 5. Targeting of pathogenic bacteria

A large numbers of in vitro studies have provided proof-of-concept that APDT kills a broad spectrum of pathogenic bacteria. Selective results on some important resistant pathogenic bacteria, both Gram-positive and Gram-negative, are presented in this section.

### 5.1. Pseudomonas aeruginosa (Gram-negative)

*P. aeruginosa* survives in both normoxic and hypoxic atmospheres and thrives in water, soils, skin, and natural and artificial environments created by humans. The organism uses a broad variety of sources for nourishment and is biologically versatile, as a result of which it can cause serious infection in diseased/damaged tissues where oxygen levels are relatively low, particularly in immunocompromised hosts (opportunistic infections). The most important problem is that *P. aeruginosa* is frequently found in hospitals on medical equipment, where it constitutes a notable source of nosocomial infections. *P. aeruginosa* has developed antibiotic resistance due to its capacity to adapt through relatively rapid planktonic→biofilm phase transition and quorum sensing [137], i.e., the capacity to change genotype based on population density. These aspects make *P. aeruginosa* a medically serious problem. A positive clinical aspect is that these infections are generally easily accessible to PSs as well as optical fibers for APDT.

The in vitro APDT efficacy using toluidine blue O (0-500 µM) was determined in multidrug resistant *P. aeruginosa* and compared to its susceptible counterpart [138]. Furthermore, the APDT efficacy was independent of efflux pump functionality or the level of resistance [25]. The authors suggested that DNA damage was the main cause of cell death, despite the fact that DNA damage is not a putative cause of APDT-mediated cell death (section 4.2). At this point, however, SOS signaling and subsequent DNA repair cannot be ruled out until this has been demonstrated in *P. aeruginosa* mutants with an impaired DNA repair machinery. Also, mechanistic studies need to be carried out to identify the localization of PSs and exact targets in *P. aeruginosa*.

In addition to toluidine blue O, APDT with the porphyrin precursor 5-aminolevulinic acid (5-ALA) was shown to be effective against *P. aeruginosa*, albeit at a relative high concentration (10 mM). This was confirmed in another study [139] that underscored that 5-ALA, and particularly its long chain derivatives, remain interesting PSs for APDT of *P. aeruginosa*. Regardless of the high dose, 5-ALA and derivatives exhibit selectivity towards *P. aeruginosa* and are harmless towards healthy surrounding tissues [140].

### 5.2. Staphylococcus aureus (Gram-positive)

Among all Gram-positive pathogens, *S. aureus* is the most extensively studied as a serious cause of life threatening infections such as skin-, soft tissue-, and blood stream infections both in hospitals and in the community. A significant reduction in MRSA viability was observed in vitro for numerous PSs, such as phthalocyanine, porphyrin, chlorines, and phenothiazinium [141-143]. Although many in vitro studies demonstrated that APDT could effectively kill *S. aureus* in either its planktonic form or in biofilms using diverse PSs, fewer reports could be found that furnished in vivo proof [144].

Some unique findings on APDT targeting *S. aureus* are noteworthy. For example, it was reported that taking advantage of the common resistance mechanism of MRSA is one of the promising strategies to improve the specificity of PSs towards MRSA [145]. A specific enzyme-activated structure (β-LEAP) was created, for which two phenothiazinium PSs (EtNBS-COOH) were combined to the side chains of cephalosporin. The two PSs were quenched in the uncleaved construct due to close proximity with each other, but were activated through cleavage of the lactam ring by beta-lactamase, which was synthesized only by resistant strains. This differs from the common targeting strategies for other bacteria (antibody conjugation, attachment of antimicrobial peptides, etc.).

A dedicated review on APDT treatment of MRSA can be found elsewhere [144].

### 5.3. Mycobacterium tuberculosis (Gram-negative)

*M. tuberculosis* is the main cause of multidrug resistant tuberculosis (MDR-TB) that emerged in the 1990s and was soon succeeded by the extremely drug resistant tuberculosis (XDR-TB). XDR-TB has developed resistance to all effective anti-TB drugs, including fluoroquinolones, and at least one of the injectable drugs (e.g., kanamycin, amikacin, capreomycin) [146].

*M. tuberculosis* possesses a distinct and rigid cell envelope structure [147]. The envelope is composed of an outer layer called ‘capsule’ [148], an outer membrane consisting of mycolic acid, and several distinctive lipids (such as trehalose monomycolate and dimycolate, phthiocerol dimycocerosate, sulpholipid-1, diacyl trehalose, and pentacyl trehalose), and an asymmetric cytoplasmic membrane [149]. This complex envelope structure makes *M. tuberculosis* a rather difficult target for APDT in term of PS uptake. However, in 1903, Niels Finsen was awarded the Nobel Prize for inventing light therapy for lupus vulgaris, which was based on the excitation of endogenous porphyrins (coproporphyrin) in *M. tuberculosis* [150].

Since then, scientists have been able to improve the APDT efficacy against this bacterium. A PS mixture called Radachlorin, containing chlorin e6, chlorin p6, and purpurin, and a PEG-conjugated pheophorbid-amethyl ester (DH-I-180-3) have been tested [151] for their APDT efficacy against clinical strains of *M. tuberculosis* isolated from patients. Repeated APDT or intermittent APDT with pulsed irradiation using a light dose of 20 J/cm^2^ exhibited better killing efficacy than APDT using continuous light irradiation. The mechanisms of cytotoxicity and the biomolecular targets were, however, not investigated.

It is noteworthy that secreted antioxidant enzymes (catalase/peroxidase and superoxide dismutase) found in the “capsule” [148] may influence the ROS-based bactericidal effect of APDT, which implies that deeper penetration and intracellular accumulation of the PS is of utter importance for killing *M. tuberculosis*. Nevertheless, encouraging findings of porin-like beta-barrel membrane proteins such as OmpATb [152] and Rv1698 [153] in the outer membrane of *M. tuberculosis*, which are proposed to transport small hydrophilic molecules [154] (albeit at significantly lower efficiency than other Gram-negative species such as *E. coli* [155]), may act as a transport channel for PSs, and cationic PSs in particular [151]. Furthermore, APDT was also shown to be effective in vitro against other *Mycobacterium* species that cause MDR-TB jointly, such as *M. smegmatis*. The administration of 7.5 μM cationic tetra-Py^+^-Me combined with illumination with a light dose of 18 J/cm^2^ reduced the viability of these bacteria by 5-6 log within 5 minutes [156].

The abovementioned in vitro data showed promising aspects of killing the mycobacteria species with APDT. It is important to note, however, that *M. tuberculosis* is an intracellular bacterium. The models for studying this species in terms of therapy should therefore ideally entail infected mammalian cells (preferably in the lungs) to achieve a representative level of clinical relevance. Although not with *M. tuberlucosis* itself, researchers conducted proof-of-concept studies using bacteria from the same family. A mouse model of *Mycobacterium bovis-*induced granulomatous infection was successfully established using collagen scaffold gel to study Verteporfin-APDT efficacy [157]. A 0.7 log bactericidal effect was found in vivo that was comparable to in vitro result using 60 J/cm^2^ light dose [157].

The fact that these bacteria thrive inside host cells is a significant therapeutic conundrum. A high level of PS targeting selectivity is exacted during APDT so that bacteria are killed and parenchymal cells are not. Although the surface of mammalian cells and bacteria is negatively charged (mammalian cells have a highly sulfated glycocalyx [3]), which is ideal for cationic PSs, the PS has to transgress at least two biomembranes to enter the bacteria. One possible strategy is to use porphyrin precursors such as 5-ALA or a hexylester derivative of ALA, which are more easily taken up due to their smaller size, and can be converted in *M. phlei* and *M. smegmatis* to coproporphyrin [158]. However when using such approach, it is important to avoid or limit phototoxicty to healthy cells because ALA can be converted to the light-sensitive protoporphyrin in mammalian cells [159].

### 5.4. Streptococcus mutans (Gram-positive)

*S. mutans* is mainly located in the mouth, where it causes biofilm formation and contributes to dental caries [160,161]. APDT is a suitable treatment modality for *S. mutans* due to accessibility of the infection site and, above all, is effective against *S. mutans* [162], at least in vitro. Nevertheless, the clinical relevance of APDT of oral *S. mutans* is less significant than other infections due to the initially non-life-threatening nature of the infection, which is characterized by biofilm formation on teeth (dental plaque). Biofilm is typically recalcitrant to conventional antibiotic treatment, which generally aims to kill pathogenic microbes or block biofilm production [163]. A common clinical application of APDT of *S. mutans* is therefore disinfection of root canals [164]. In addition to their cariogenicity, *S. mutans* has been implicated in more severe conditions, including cerebral microhemorrhage [165] and infection of heart valves as well as atheromatous plaques in blood vessels [166]. At this point, PDT has been investigated for the treatment of atheromatous plaque, albeit without direct targeting of *S. mutans*.

With respect to APDT efficacy, Rose Bengal (0.5 µM) (section 2.1) caused complete eradication of planktonic *S. mutans* (3 Log10 CFU/mL) following irradiation with 400-600-nm light [162]. Another in vitro study reported that *S. mutans* biofilm was reduced by 0.62 log CFU/mL by Rose Bengal (5 μM) combined with blue LED irradiation (455 ± 20 nm) [167]. The lower killing efficacy in the latter study [167] was likely caused by the complex nature of biofilm, which is composed of different pathogens, compared to the homogeneous culture of *S. mutans* [161]. APDT of *S. mutans* biofilm with erythrosine at the same concentration (5 μM), a clinically approved PS for dental plaque removal, yielded slightly better results in terms of cell killing capacity [168]. Moreover, APDT with toluidine blue O (section 2.1) resulted in the eradication of *S. mutans* in 10 days-old biofilm [169]. Toluidine blue O exhibited no dark toxicity and the efficacy was light dose-dependent. Finally, curcuminoids (section 2.5.2) may be suitable PSs for APDT of *S. mutans* in that curcuminoids exert dual pharmacodynamic activity. On the one hand, curcuminoids block sucrose-dependent adherence of *S. mutans* to saliva-coated hydroxyapatite discs (model for teeth) and inhibit the acidogeni-city and aciduricity of *S. mutans* biofilms [170]. On the other hand, APDT of bacteria photosensitized with curcumin induces cell death [95]. The encouraging results notwithstanding, these in vitro results need further in vivo validation.

### 5.5. Enterococcus faecalis (Gram-positive)

*E. faecalis* mainly resides in the gastrointestinal tract but also occurs in root canals with persistent endodontic infections. The species is resistant to conventional antibiotic treatment [171] and is responsible for causing nosocomial infections.

Several in vitro studies explored the possibility of eliminating planktonic *E. faecalis* as well as its biofilm with APDT using cationic PSs such as eosin Y [172], methylene blue [173], tetra-Py^+^-Me [174], and SAPYR (2-((4-pyridinyl)methyl)-1Hphenalen-1-one chloride) [174,175]. APDT of root canal specimens prepared from extracted teeth infected with *E. faecalis* achieved bacterial reduction of 77.5% when methylene blue (6.5 µg/mL) and 60 J/cm^2^ light dose (665 nm) were applied [173]. A considerable bacterial reduction (40.5%) was also achieved in the control group with only light treatment [173], suggesting that the species may produce its own PSs or is otherwise sensitive to red light. In another study, the efficacy of continuous, repeated, and intermittent APDT with eosin Y as PS was compared [172]. It was found that higher dose of PS (40 or 80 µM) during continuous or intermittent APDT did not increase the bactericidal effect against *E. faecalis* biofilm, which was possibly due to aggregation of the PS at high concentration upon irradiation. In contrast, repeated exposure APDT (a constant irradiation time of 960 s with 10-s pauses, 10 or 20 µM of eosin Y), the extent of bacterial cell death was 96% [172]. Cieplik et al. [174] compared the photodynamic efficacy of the new generation PS SAPYR and tetra-Py^+^-Me in *E. faecalis*. The study revealed that the absolute ^1^O_2_ singlet quantum yield of SAPYR was about 1/5 of that of tetra-Py^+^-Me due to lower photon absorption. However tetra-Py^+^-Me had no effect against *E. faecalis* while SAPYR eliminated the pathogen by 5 logs [174] in both monospecies and polyspecies biofilm. These results collectively show the promising prospects of APDT against *E. faecalis* in endodontic infections.

### 5.6. Helicobacter pylori (Gram-negative)

*H. pylori* resides in the human gastric tract and causes infections that lead to peptic ulcers and gastric cancer [176]. Conventional treatment of *H. pylori* infections includes the administration of antibiotics together with proton pump inhibitors [177], which often causes undesired side effects such as epigastric pain, nausea, and diarrhea [177]. Resistance of *H. pylori* to different antibiotic regimens including amoxicillin, clarithromycin, metronidazole, levofloxacin, tetracycline, and rifampicin has been reported in regional studies from different countries [178-181] and is particularly causing chronic health problems in children [182]. Consequently, APDT has been employed as a possible, more patient-friendly alternative to conventional therapy [183-185].

Although the stomach is an internal organ, accessibility for PSs (oral administration) and laser probes (e.g., endoscopically) are not expected to be clinical bottlenecks. In fact, APDT of *H. pylori* does not necessarily have to rely on external PS delivery inasmuch as the bacteria naturally produce porphyrins, such as protoporphyrin and coproporphyrin, via the heme biosynthesis pathway [185]. Accordingly, laser irradiation with blue light (~405 nm) without prior photosensitization of cultured *H. pylori* is sufficient to induce cell death [185]. A clinical study using 405-nm endoscopic light confirmed APDT efficacy in eradicating *H. pylori* colonies in patients [186]. In this small scale pilot study, 9 eligible patients received endoscopic blue light at a fluence of 40 J/cm^2^. No administration of PS was necessary as *H. pylori* was able to naturally synthesize protoporphyrin IX and coproporphyrin. Consequently, 99% bacterial inactivation was achieved. However the exact mechanism underlying the photo-inactivation of *H. pylori* is yet to be determined.

## 6. Optimization of photodynamic efficacy

Presently, a major limitation of APDT is the inadequate uptake of PS by bacteria and hence insufficient photosensitization to induce lethality. Studies have explored various means to optimize the selective uptake of PSs by bacteria, which include structural modifications of existing PSs, including conjugation of anionic/neutral PSs to cationic polymers/surfaces [187-189] and the development of novel PSs [7,190]. Some alternative methods are discussed next in addition to those already addressed (e.g., chemical perturbation of biofilm (section 2.5.1)).

### 6.1. Liposomal photosensitizer delivery systems

Liposomes are nanoscopic fat droplets composed of a phospholipid bilayer and an aqueous core. Consequently, liposomes can encapsulate both hydrophilic and lipophilic PSs [32] for delivery into bacteria [191]. In fact, liposomes have been recommended for these purposes due to their fluidic lipophilic nature and the possibility to bear a positive surface charge

[191]. Cationic carriers can disorganize the native three-dimensional architecture of the bacterial cell envelope, thereby inducing membrane permeability and easier transmembrane passage or settlement of the PS [192]. The increased cytotoxicity of liposome-delivered PS relative to control delivery (unencapsulated PS) may also be ascribed to the more suitable localization of the PS in the cell envelope [191,192]. Proof-of-principle for liposomal PS delivery has been provided in methicillin-resistant *S. aureus* and *P. gin- gialis*, where highly cytotoxic APDT was achieved with cationic liposome-delivered ZnPc [193]. Tsai et al. [194] reported that APDT with liposomal hematoporphyrin improved therapeutic efficacy in gram-positive pathogens such as MSSA, MRSA, *Staphylococcus epidermidis*, and *Streptococcus pyogenes*. The development and in vitro proof-of-concept of ZnPc-containing cationic liposomes (section 2.3) has also been described in the context of PDT of cancer cells [32,195,196]. These formulations may therefore be useful for APDT.

### 6.2. Conjugation of cationic antimicrobial peptides to photosensitizers

Cationic antimicrobial peptides (CAMPs) such as buforin, magainin, and apidaecin are useful antimicrobial peptides that may be conjugated to PSs to improve PS-cell association or intracellular PS delivery [43,45]. The peptides possess bacterial lytic properties and have particular affinity for Gram-negative bacteria. The conjugation of these peptides to the PS also broadens the therapeutic spectrum of certain PSs that are initially taken up by only Gram-positive species, including 5(4’-carboxyphenyl)-10,15,20-triphenylporphyrin [43].

Another clinically important aspect of peptide conjugation is target selectivity. Since the surface of mammalian cells is also negatively charged, application of any cationic PS or any PS encapsulated in cationic liposomes may target the PS to the neighboring healthy cells and cause side effects. Conjugation of antimicrobial peptide to cationic (encapsulated) PSs [123,197,198] can therefore improve the selectivity towards bacteria and reduce the extent of uptake by host cells.

In terms of APDT efficacy, an LPS-neutralizing peptide YI13WF (YVLWKRKRKFCFI-amide) conjugated to protophophyrin IX was shown to have high killing potential in *E. coli* and *Klebsiella pneumoniae* [199]. Since protoporphyrin is taken up by mammalian cells as well, some photosensitization of non-target cells is expected. However the authors demonstrated that the dimeric conjugate exhibited higher selectivity towards bacterial cells than the protoporphyrin IX administered alone by co-incubating JurKat T cells with *E. coli* or *K. pneumoniae* during the APDT. This is probably due to the higher binding affinity of the conjugate towards the endotoxin.

### 6.3. Efflux pump inhibitors

Efflux pumps play an important role in bacterial drug resistance [200]. These pumps can either export selective anti-microbial compounds or expel a collection of multiple different compounds with diverse structures. Several PSs, such as the amphiphilic cationic phenothiazinium, are substrates of multidrug resistance efflux pumps [25] and may therefore be removed from the intracellular environment before APDT. Hence, inhibition of efflux pump function before photosensitization may improve APDT outcome, as has been demonstrated in several studies [27,28].

Specific export pump inhibitors were combined with several PSs, both cationic (methylene blue) and anionic (Rose Bengal), and evaluated in APDT of biofilm-derived *Enterococcus faecalis* [28]. APDT with methylene blue was considerably more cytotoxic to *E. faecalis* compared to Rose Bengal, and the combination with EP inhibitor verapamil hydrochloride significantly enhanced APDT-induced cytotoxicity by 3 Log at a relatively low light dose (5 J/cm^2^). Another study [27] showed that the order in which EP inhibitors (the diphenyl urea INF271, reserpine, 5'-methoxyhydnocarpin, and the polyacylated neohesperidoside, ADH7; all inhibit *S. aureus* NorA) and PS (toluidine blue O) were applied governs the bacterial killing efficacy. When the inhibitor was applied before the PS, a superior outcome was achieved. The authors contended that simultaneous inhibitor and PS application or administration of the PS before the inhibitor would cause competitive binding to NorA and hence reduce intracellular PS retention.

Despite the potential of this combinatorial regimen in APDT, no clinical feasibility studies have yet been performed, mainly due to the biological instability, poor solubility, and in vivo toxicity of some export pump inhibitors [201]. These factors, however, do not rule out the utility of this approach for the treatment of local infections, such as those that cause dental diseases and in superficial skin wounds, where systemic toxicity issues can be avoided.

### 6.4. Electroporation

Electroporation is a well-established molecular biology technique used to introduce chemicals, drugs, or DNA into the cell by transiently permeating the cell membrane with a lowvoltage electrical current [202]. It is also used clinically to treat solid tumors [203], in which case the aim is to use high-voltage electrical current to irreversibly permeate and kill cells.

Electroporation was applied to both *S. aureus* and *E.coli* cells to induce pore formation in the outer membrane and increase the delivery of hypericin into the cells [204]. This study showed that APDT/electroporation inactivated 3.67 log more *E. coli* and 2.65 log more *S. aureus* than APDT alone.

Although no in vivo studies have been performed to date with respect to electroporation in combination with APDT, such a modality has been reviewed in the context of solid tumors [205]. It was summarized in this review that electroporation typically improved the PDT effect in vitro by a factor of 2 to 5, depending on the PS, cell line, and electric field conditions. In vivo experiments showed that, when PDT is combined with electroporation, the required effective drug dose is lower and the drug-light interval is shorter than PDT alone. This reflects the potential utility of electroporation as an adju-

vant step before APDT to optimize bacterial photosensitization. It should be noted that electroporation of PSs could increase the risk of non-specific photosensitization of host cells, al-though this statement warrants experimental evidence.

### 6.5. Light source

Various light sources can be used to activate the PS, ranging from lasers and narrow-band LED sources to high-intensity broadband light sources [132]. Many factors such as the optical penetration depth of excitation light, wavelength, fluence, and drug-light interval have an impact on the photodynamic efficacy [206]. For dental infections and infected superficial wounds these factors do not weigh too heavily. For internal infections, however, sufficient light delivery to the entire infection site may be a challenge [13].

Yin et al. [207] used upconverting nanoparticles that can absorb near-infrared light (980 nm), which lies in the so-called therapeutic window for (A)PDT [32], and convert it to shorter-wavelength, high-energy photons to induce a photodynamic effect. Inasmuch as there are no endogenous chromophores that significantly absorb near-infrared light of this wavelength, the optical penetration is sufficient to homogenously irradiate larger bulks of tissue. The drawback of this technique is that another exogenous pharmacological entity (i.e., upconverting nanoparticles) must be co-administered with the PS, which may lead to systemic or local toxicity issues [208].

## 7. In vivo and clinical status quo of antibacterial photodynamic therapy

Numerous in vitro studies corroborate the efficacy of APDT in a large variety of bacteria. Although these studies are useful in providing initial proof-of-concept and studying intracellular pharmacokinetics and PS pharmacodynamics, they do not provide a solid translational basis for the clinical setting. In that respect, in vivo studies represent higher-level data.

The PSs that have been tested in vivo include phenothiaziniums (section 2.1) such as methylene blue [19,209], Rose Bengal [210], and EtNBS [188] (Table 1), ZnPc derivative RLP068/Cl [211], (Table 1), and C70 fullerene [60,64,68] (Table 1), among others. Animal models have been developed that mimic clinical infections, including a mouse model of skin abrasion caused by MRSA [212] and a guinea pig model of burn infections with *S. aureus* [213]. Moreover, a study compared the efficacy of APDT as an adjuvant treatment to scaling and root planning (SRP) of periodontitis in nicotine-modified rats [214]. Their results showed lower bone loss when toluidine blue O-APDT was applied in combination with SRP versus SRP alone, indicating that APDT was an effective adjunctive treatment to SRP for periodontitis. With respect to infected burn wounds, a mouse model [215] was established to study APDT efficacy in burn wounds infected with drug-resistant *P. aeruginosa* (section 5.3). The bacterial count in the bloodstream (sepsis) was measured after APDT with hypocrellin B:lanthanum (HB:La^3+^). APDT not only reduced the degree of the infection but also successfully lowered the bacterial count in the bloodstream by 2-3 log CFU/mL compared to the control group. This indicated that APDT could delay bacteremia upon infection. The scope of this study therefore could be broadened in the sense that APDT could be applied locally to achieve control of bacterial levels in patients’ bloodstream to improve the efficiency of certain medical treatments, provided that sepsis has been a major concern in the hospital [216,217].

During in vivo APDT experiments, animals are often sacrificed following the intervention to determine the standard bacterial count (CFU/mL). Recent developments in these models entail the inoculation of the animals with endogenously luminescent bacteria so that the animals can be followed in real time after APDT [210]. The intensity of luminescence can be directly correlated to the extent of the infection [210,218]. Genetically modified *E. coli* and *P. aeruginosa* via transformation of a plasmid containing the *P. luminescens* lux operon could stably generate bioluminescence [210].

Clinically, APDT of dental disease is the most commonly employed APDT application in patients due to easy accessibility of the infection site [219]. Tooth decay and periodontitis caused by biofilm infection that encompasses different pathogens warrant the use several different antibiotics [220]. Consequently, drug resistance is easily developed as a result of the often sublethal concentration of antibiotics in the sulcus fluid and the chronic use of antibiotics [221-223]. In those cases, APDT is considered an ideal alternative treatment [162,168].

In the past two years, more than five clinical studies have been conducted on the utility of APDT in oral infections [224-228]. Only one of the five studies reported no significant improvement in periodontal disease in smokers following APDT in conjunction with scaling and root planning [226], although good results had been obtained in animal studies [214]. The other four studies provided compelling support for the bactericidal efficacy of APDT in localized dental infectious diseases [224-228]. Corroboratively, a recent systematic review yielded favorable results in regard to APDT for the treatment of infected root canals and called for a well-established clinical protocol [229]. Moreover, APDT has been suggested as an adjunctive treatment after standard endodontic treatment [225].

Following the developments in clinical oral diseases, APDT is progressively becoming an acceptable treatment modality for chronic and drug-resistant infections [13,22,162,183,230]. A PS, PPA904, was evaluated in a phase II clinical trial for the treatment of chronic leg ulcer induced by MRSA and yielded good bactericidal results. APDT with PPA904 significantly reduced the number of patients (18%) who suffered from symptoms of infection post-APDT compared to patients who received placebo treatment [231]. Another recent clinical study reported that APDT of *P. aeruginosa*-caused skin ulcers in the lower limbs resulted in significant bactericidal outcome and wound healing-promoting effects [232]. This is in line with a previous study in mice, where APDT was shown to reduce the hyper-inflammatory response in *P. aeruginosa*-infected skin wounds [233]. Additionally, APDT is also used in the clinical management of diabetic foot, which causes sizeable infection-related complications in diabetic patients. One clinical study reported that 17 of the 18 diabetic foot patients who received antibiotics plus PDT did not have to undergo limb amputation, whereas all the patients who received antibiotics only (N = 16) were subjected to limb amputation [234]. Due to the limited cohort size and the lack of randomization and doubleblind conditions, the application of APDT in diabetic patients requires more robust clinical evidence.

Besides the curative prospects of APDT, Huang et al. [235] reviewed the encouraging preventative effects of (A)PDT in the context of the clinically approved Photofrin. (A)PDT can trigger a host immune response even when applied before infections take place, which in turn may contribute to the prevention and treatment of bacterial arthritis. Accordingly, preventive immunomodulation is a novel field that could be employed to control the degree of infection by pre-emptively priming the host’s immune system.

## 8. Adaptive mechanisms in bacteria and therapeutic recalcitrance

To date the putative contention is that bacteria do not develop resistance against APDT, as no resistance has been reported among any bacterial species. Failure of some PSs to effectively kill certain bacterial species is due to delivery challenges and not necessarily the result of resistance. Some PSs are substrates of multidrug efflux pumps, as mentioned in section 2.1, and may therefore be eliminated from the target cells as part of bacterial resistance. Concomitant administration of pump inhibitors can counteract these resistance mechanisms.

Tim Maisch [230] recently described possible routes for bacteria to develop resistance towards APDT, which includes overexpression of antioxidant enzymes and/or heat shock proteins to protect bacteria from the post-APDT oxidative stress. However, APDT-induced oxidative stress occurs very rapidly upon illumination, and bacteria are unlikely to acquire resistance in such a short time. Also, since ROS have very short lifetimes in biological environments, it is questionable whether the synthesis of antioxidants or the transcription and translation of antioxidant enzymes in response to the acute oxidative stress (a process that takes considerably longer) is effective. Furthermore, no defense system against singlet oxygen has been found so far among bacterial pathogens, pleading for the use of type II PSs. Nevertheless, APDT-related resistance issues seem to be a minor concern at this point.

## 9. Conclusions

APDT is considered a promising means to overcome the difficulties in treating infections caused by multidrug-resistant bacteria.

The complex structure of the bacterial cell envelope, and especially that of Gram-negative species, is the main challenge for both conventional treatment and APDT. Although it has been suggested that uptake of PSs is not required per se to kill bacteria by APDT, selective PS binding and passage through the bacterial cell envelope is favored. The photodestructive effect of ROS at multiple intracellular targets, including the membrane, is unequivocally the biggest advantage of APDT over conventional antibiotic approaches, which often comprise a single target. Several measures are available to optimize intracellular delivery, which range from functionalization of PSs with cationic moieties to pharmacological interventions to electroporation and highly technological means of PS excitation.

Undoubtedly, APDT is still in its infant stage of development, but nevertheless harnesses clinical potential in light of failing conventional treatments. The pharmacodynamics, pharmacokinetics, and disposition of PSs, the drug-light intervals and laser settings, and the biology of the infection site dictate clinical outcome of APDT treatment. In order to implement APDT in the clinical setting, most of these parameters still need to be determined for the majority of indications. A lot of in vitro proof-of-concept work is available. The next phase should be in vivo validation of the in vitro findings and small clinical proof-of-concept studies in order to push this technology closer to actually benefitting patients with resistant infections.
